# Mesoporous Cobalt Oxide (CoO_x_) Nanowires with Different Aspect Ratios for High Performance Hybrid Supercapacitors

**DOI:** 10.3390/nano13040749

**Published:** 2023-02-16

**Authors:** Haomin Ji, Yifei Ma, Zhuo Cai, Micun Yun, Jiemin Han, Zhaomin Tong, Mei Wang, Jonghwan Suhr, Liantuan Xiao, Suotang Jia, Xuyuan Chen

**Affiliations:** 1 State Key Laboratory of Quantum Optics and Quantum Optics Devices, Institute of Laser Spectroscopy, Collaborative Innovation Center of Extreme Optics, Shanxi University, Taiyuan 030006, China; haominji@126.com (H.J.); caizhuo2229@163.com (Z.C.); yunmicun@163.com (M.Y.); jiemin.han@foxmail.com (J.H.); zhaomin.tong@sxu.edu.cn (Z.T.); xlt@sxu.edu.cn (L.X.); tjia@sxu.edu.cn (S.J.); xuyuan.chen@usn.no (X.C.); 2 Department of Polymer Science and Engineering, School of Mechanical Engineering, Sungkyunkwan University, Suwon 16419, Republic of Korea; suhr@skku.edu; 3 Faculty of Technology, Natural Sciences and Maritime Sciences, Department of Microsystems, University of Southeast Norway, N-3184 Borre, Norway

**Keywords:** CoO_x_ nanowires, aspect ratios, capacity, hybrid supercapacitors

## Abstract

Cobalt oxide (CoO_x_) nanowires have been broadly explored as advanced pseudocapacitive materials owing to their impressive theoretical gravimetric capacity. However, the traditional method of compositing with conductive nanoparticles to improve their poor conductivity will unpredictably lead to a decrease in actual capacity. The amelioration of the aspect ratio of the CoO_x_ nanowires may affect the pathway of electron conduction and ion diffusion, thereby improving the electrochemical performances. Here, CoO_x_ nanowires with various aspect ratios were synthesized by controlling hydrothermal temperature, and the CoO_x_ electrodes achieve a high gravimetric specific capacity (1424.8 C g^−1^) and rate performance (38% retention at 100 A g^−1^ compared to 1 A g^−1^). Hybrid supercapacitors (HSCs) based on activated carbon anode reach an exceptional specific energy of 61.8 Wh kg^−1^ and excellent cyclic performance (92.72% retention, 5000 cycles at 5 A g^−1^). The CoO_x_ nanowires exhibit great promise as a favorable cathode material in the field of high-performance supercapacitors (SCs).

## 1. Introduction

The urgent need for power sources in the power grid has driven extensive research on novel energy storage devices [1,2,3,4,5]. SCs with rapid discharge and broad working temperature range are widely studied as green energy storage devices [6,7]. Electric double-layer capacitors utilize a process of physical adsorption and desorption, in which carbon materials (graphene, carbon nanotubes) are generally used as active materials, which are characterized by long lifetimes but low specific energy [8]. A pseudocapacitor, based on the mechanism of the redox reaction of the electrode, is a novel energy storage device that is expected to hold both high specific energy and specific power. Among the cathode materials, the transition metal oxides (represented by Co_3_O_4_) have obtained the most extensive research because of multiple valence states and high theoretical specific capacity [9]. Electrodes based on various morphologies and sizes of Co_3_O_4_ have been studied recently. For instance, Zhou et al. synthesized ultrathin Co_3_O_4_ nanosheets@Ni foam that presented an excellent gravimetric capacity of 882 C g^−1^ [10]. By a facile hydrothermal procedure, Liu et al. stated a synthesis of Co_3_O_4_ mesoporous nanospheres with homogeneous core-shell structure, which possessed a gravimetric capacity of 335 C g^−1^ [11]. Mahesh et al. synthesized porous Co_3_O_4_ nanowire arrays by a precipitation method, and the electrode exhibits a low equivalent series resistance of 0.4 Ω and a superb gravimetric capacity of 1004 C g^−1^ [12]. By comparison, it is found that Co_3_O_4_ nanowires obtain the highest pseudocapacitive performances due to their abundant contact sites, which are more conducive to ion transport and electron conduction [13,14]. However, the length and diameter of the Co_3_O_4_ nanowires vary widely, and the overall morphology of the nanowires is also contrary in related studies [15,16,17,18]. Therefore, the relationship between the aspect ratio of Co_3_O_4_ nanowires and the capacity performance still needs to be comprehensively studied [19].

In addition, compositing with conductive materials is an effective strategy to enhance the energy storage capability of pseudocapacitive materials. For example, compositing with low-dimensional materials (graphene, MXene, etc.) can ameliorate the inherent electric conductivity and volume changes in redox reactions of the Co_3_O_4_ [20]. In our previous work, we synthesized holey graphene-coated Co_3_O_4_ cubes and obtained a gravimetric capacity of 1085.6 C g^−1^, which is notably higher than that of pure Co_3_O_4_ cubes [21]. Nonetheless, these 2D materials usually have a low specific capacity, and the decrease in Co_3_O_4_ content will inevitably result in a decrease in the actual specific capacity of active materials [22,23,24]. Extensive work has shown that superior specific capacity can be obtained by combining Co_3_O_4_ with CoO, which is usually ascribed to the following reasons: (1) Both Co_3_O_4_ and CoO have high theoretical specific capacities (1602 and 1931 C g^−1^) [25]; (2) The Co_3_O_4_/CoO composites exhibit similar redox reactions under corrosive alkaline conditions, which can contribute to enhanced electrochemical properties [26,27]. For example, Cheng et al. developed mesoporous microspheres composed of CoO and Co_3_O_4_ with a core-shell structure, which exhibited an impressive specific capacity of 1520 C g^−1^ [28]. Pang et al. synthesized porous mixed-phase CoO/ Co_3_O_4_ composites, which delivered a low equivalent series resistance of 0.16 Ω, and the capacity retained 108% after 5000 cycles [29]. However, there have rarely been reported on the Co_3_O_4_/CoO nanowires as active materials for SCs applications.

In this paper, self-supported CoO_x_ nanowires are synthesized by facile hydrothermal reaction and annealing treatment. By controlling the hydrothermal temperature to affect the reaction rate and the anisotropy of nanowires, nanowires with different aspect ratios were obtained, forming different microscopic morphologies. As SCs electrodes, CoO_x_ nanowires exhibit an ultra-high mass specific capacity and can operate at 100 A g^−1^. To further explore the practical application of CoO_x_ nanowires electrodes, HSCs composed of CoO_x_ nanowires and commercial AC electrodes exhibit high specific energy, superb rate performance, and outstanding cyclic stability.

## 2. Materials and Methods

### 2.1. Preparation of Self-Supported CoO_x_

Before synthesizing CoO_x_ nanowires, nickel foam (NF, 1 × 1.5 cm^2^, 0.5 mm thick) was successively sonicated (40 Hz, 99%) in dilute hydrochloric acid (10 wt%), alcohol (AR), and DI-water (σ = 18.25 MΩ·cm) for 30 min to clear off the oxides and organic residues. The self-supported CoO_x_ nanowires were acquired via a hydrothermal reaction combined with a subsequent calcination treatment. The hydrothermal reaction solution was prepared by dissolving 2 mmol of cobalt nitrate (Co(NO_3_)_2_·6H_2_O, AR) and urea (CO(NH_2_)_2_, AR) powder into 35 mL DI-water in turn. After stirring to completely dissolve the powders, an orange-red solution was obtained. Put two pieces of dried NF in the aforementioned solution and kept at room temperature for 12 h, then transferred into a Teflon-lined stainless-steel autoclave (50 mL). The hydrothermal process was implemented in a vacuum oven at 110, 120, 130, and 140 °C for 10 h. Precursors were grown on NF after the hydrothermal process and rinsed subsequently with DI-water. After vacuum drying (60 °C, 24 h), the dried NF was annealed with a heating rate of 5 °C min^−1^ (350 °C, 2 h, Ar atmosphere) in a tubular furnace to obtain black CoO_x_ nanowires grown on the NF. After the tubular furnace was naturally cooled to room temperature, the electrode was obtained, which could be directly used for electrochemical testing. The CoO_x_ nanowires obtained at the hydrothermal temperature of 110, 120, 130, and 140 °C were named CoO_x_-110, CoO_x_-120, CoO_x_-130, and CoO_x_-140, respectively. The mass loadings of CoO_x_-110, CoO_x_-120, CoO_x_-130, and CoO_x_-140 were 1.7, 1.45, 1.23, and 0.74 mg, respectively.

### 2.2. Material Characterizations

Field emission scanning electron microscopy (FE-SEM, HITACHI SU-8010, Tokyo, Japan) was implemented to observe the morphologies at magnifications of 5−100k. X-ray diffraction (XRD, Empyrean, Beijing, China) was used to observe the phase structures under Cu Kα radiation at the angle of 5−90° with a speed of 2° min^−1^. X-ray photoelectron spectrometry (XPS, Thermo ESCALAB 250XI, Massachusetts, USA) was used to analyze the elemental compositions. The N_2_ adsorption-desorption isotherm at 77 K was tested using a MicrotracBEL BELSORP-max (Osaka, Japan) specific surface area analyzer to analyze the surface areas and pore distributions.

### 2.3. Electrochemical Measurements

An electrochemical workstation (Bio-Logic, VSP-300, Grenoble, France) was used for all electrochemical tests at room temperature. The electrode test was carried out where the CoO_x_ nanowires on NF, Ag/AgCl electrode (ϕ = 6 mm), and platinum sheet (1 × 1 cm^2^, 0.1 mm thick) were applied as working, reference, and counter electrodes, respectively. The 2 M KOH (AR, 90%) solution was used as the electrolyte.

Gravimetric specific capacities (Q_g_, C g^−1^) were calculated based on the galvanostatic charge/discharge (GCD) and cyclic voltammetry (CV) curves using Equations (1) and (2), respectively:

Q_g_ = t × I/m, (1)

Q_g_ = ∫IdV/2mν, (2)

where the t and I/m are the discharge time (s) and current density (A g^−1^); ∫IdV, m, and ν are the area of the CV curves, mass loading of CoO_x_ (mg) and scan rate (mV s^−1^), respectively.

The HSC was composed of CoO_x_ cathode and AC anode. AC slurry was prepared by mixing AC (2100 m^2^ g^−1^), carbon black (CB, 62 m^2^ g^−1^), and polyvinylidene (PVDF, M_w_~400,000) difluoride in N-methylpyrrolidone (NMP, 99%) with a gravimetric ratio of 8:1:1. NF was used as the current collector. The optimal ratio of the m (CoO_x_) to m (AC) is determined by Equation (3) based on charge balance theory.

m^+^ × Q_g_^+^ = m^−^ × Q_g_^−^, (3)

The gravimetric specific capacity is calculated by Equation (1) using GCD data, where the m is the total mass of the CoO_x_ cathode and AC anode. The specific energy (E_c_, Wh kg^−1^) and specific power (P_c_, W kg^−1^) were calculated using Equations (4) and (5), respectively.

Ec = Qg × ΔV/7.2,(4)

Pc = 3600 Ec/t,(5)

where the ΔV is the working window of the HSC (V).

## 3. Results and Discussion

**Figure 1:**
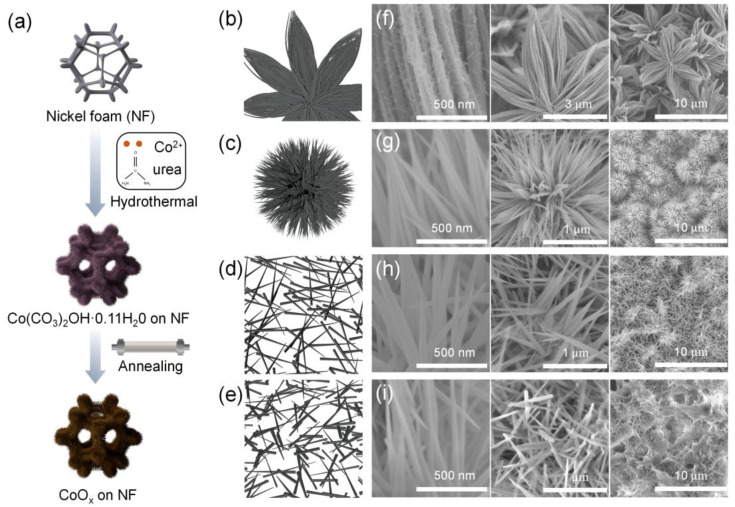
(**a**) Schematic illustration of the fabrication process of the self-supported CoO_x_; schematic diagrams of the (**b**) CoO_x_-110, (**c**) CoO_x_-120, (**d**) CoO_x_-130, and (**e**) CoO_x_-140; FE-SEM images of the (**f**) CoO_x_-110, (**g**) CoO_x_-120, (**h**) CoO_x_-130, and (**i**) CoO_x_-140.

Self-supported CoO_x_ nanowires on NF are prepared by a simple hydrothermal reaction followed by a post-annealing procedure, as illustrated in Figure 1a. Disparate hydrothermal temperatures will lead to disparate rates and degrees of reaction, resulting in different aspect ratios and structures of the nanowire arrays. The nanowire array structures of CoO_x_ under four conditions show differently, as displayed in Figure 1b−e. The surface morphology and structural characteristics of CoO_x_ are observed by FE-SEM. In Figure 1f, the CoO_x_-110 displays a nanoflower structure at a low magnification, and the nanowires are about 5 μm in length and 50 nm in diameter at a high magnification. As the reaction temperature increased to 120 °C, the diameter of the CoO_x_-120 nanowires is still about 50 nm, but the length becomes 1−2 μm, showing an urchin-like structure clearly (Figure 1g). When the hydrothermal temperature is raised to 130 °C, the morphology changes to a tiled grass-like structure (Figure 1h) with a larger exposed surface area, which is favorable to the infiltration of electrolyte and ion transport, thereby leading to excellent electrochemical properties [30]. Compared with the uniform distribution of the CoO_x_-130 nanowires, the CoO_x_-140 nanowires have shorter lengths and obvious structural damage, which may inevitably yield a decline in electrochemical performance (Figure 1i).

**Figure 2:**
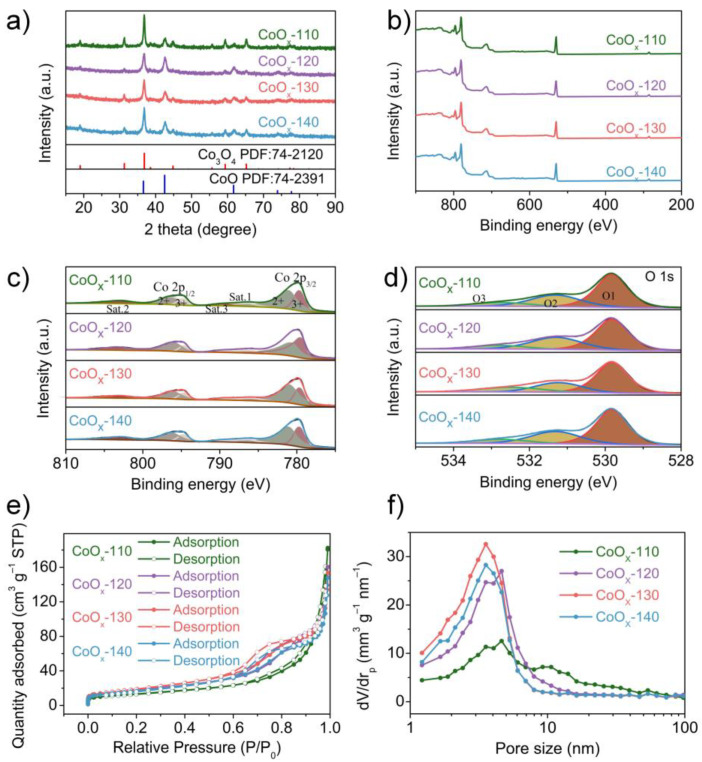
(**a**) XRD pattern, (**b**) XPS survey spectrum, high-resolution XPS spectra for the (**c**) Co 2p, and (**d**) O 1s, (**e**) N_2_ adsorption and desorption isotherms, and (**f**) pore size distributions of the CoO_x_-110, CoO_x_-120, CoO_x_-130, and CoO_x_-140 specimens.

To confirm the crystallographic structures of the as-synthesized nanowires, the specimens are analyzed by XRD Corresponding to the (111), (220), (311), (222), (400), (422), (511), and (440) planes of the cubic crystal structure Co_3_O_4_ (JCPDS no.74−2120), diffraction peaks are observed at 2θ of 18.99, 31.27, 36.84, 38.54, 44.80, 55.65, 59.35, and 65.23° in Figure 2a. Besides, several obvious peaks at 36.59, 42.50, 61.67, 73.89, and 77.78° are also detected, which can be indexed to CoO (JCPDS No.74−2391). These results confirm that the obtained CoO_x_ nanowires are hybrids of Co_3_O_4_/CoO, and the mass proportion of the Co_3_O_4_ and CoO in CoO_x_ nanowires are obtained by quantitative phase analysis, as shown in Table S1. Moreover, the XRD pattern of the precursor of CoO_x_-130 is also characterized to further explore the reaction process, and the result is shown in Figure S1. Multiple diffraction peaks indicate that the composition of the precursor is complex, but the main component is certainly Co_6_(CO_3_)_2_(OH)_8_·H_2_O (JCPDS No.48–0083) [31]. Accordingly, the chemical reactions related to the precursors can be described as follows:

6Co^2+^ + 8OH^−^ + 2CO_3_^2−^ + H_2_O → Co_6_(CO_3_)_2_(OH)_8_·H_2_O

In addition, the elemental and valence composition of the prepared CoO_x_ nanowires are described in detail by XPS to further determine the sample composition. In Figure 2b, the signals of Co 2p, O 1s, and C 1s appear in XPS survey spectra, indicating the existence of Co, O, and C elements in all samples. Three satellites and two spin-orbit doublets are considered to fit the high-resolution spectrum of Co 2p in Figure 2c. Two intense satellite peaks (Sat. 1 and Sat. 2) at 786.5 and 803.1 eV indicate abundant Co^2+^, demonstrating the existence of CoO [32]. Moreover, Co_3_O_4_ is also presented in the CoO_x_ according to distinguishable Sat. 3 peak at 789.5 eV, which indicates the existence of Co^3+^. Furthermore, the fitting peaks at 780.9, 796.3 eV, and 779.6, 794.7 eV are corresponded to the bivalent and trivalent cobalt, respectively [33,34]. In Figure 2d, three peaks (O1, O2, and O3) were fitted in accordance with the high-resolution O 1s spectrum. The fitting peaks of O1, O2, and O3 at 529.8 eV, 531.4 eV, and 532.6 eV are related to the O elements in CoO_x_, surface H_2_O, and oxygen vacancies, respectively [35,36]. The spectral results confirm that the CoO_x_ samples are Co_3_O_4_/CoO composites, in agreement with the results from XRD.

The pore size of the electrodes will affect the infiltration of electrolytes, which is one of the indispensable characteristics of the SC electrodes. Therefore, the specific surface area and pore size distribution of the CoO_x_ electrode are evaluated by testing N_2_ adsorption and desorption isotherms (Figure 2e). All CoO_x_ samples show type-IV isotherms, confirming the presence of mesopores, which are the most favorable aperture sizes for SCs application. According to the BET analysis, the specific surface area of the CoO_x_-130 is 66.64 m^2^ g^−1^, which is higher than that of CoO_x_-110, CoO_x_-120, and CoO_x_-140 (47.2, 66.04, and 58.3 m^2^ g^−1^). The average pore sizes of CoO_x_ obtained by BJH analysis are 23.89, 16.52, 14.24, and 15.71 nm, which is consistent with the type-IV isotherms. The large specific surface area and small mesoporous distribution of the CoO_x_-130 facilitate full contact between CoO_x_ nanowires and KOH electrolytes, which can enable fast ion transport and electron conduction, thereby improving the electrochemical properties of the CoO_x_-130 [22,37].

**Figure 3:**
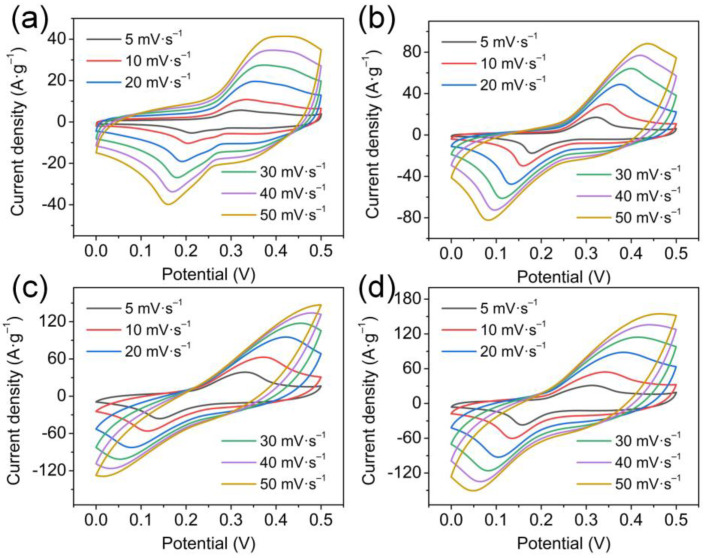
CV curves of (**a**) CoO_x_-110, (**b**) CoO_x_-120, (**c**) CoO_x_-130, and (**d**) CoO_x_-140 at various scan rates.

After determining the successful preparation and obtaining the pore size information of the CoO_x_ nanowires, the electrodes are applied to SCs as cathodes in a 2 M KOH electrolyte. In Figure 3a−d, different from the rectangular CV curves of capacitive materials, all CoO_x_ electrodes exhibit obvious symmetry redox peaks at various scan rates, belonging to the typical electrochemical features of battery-like materials. The possible redox reactions have been reported in the recent literature [20,38,39].

**Figure 4:**
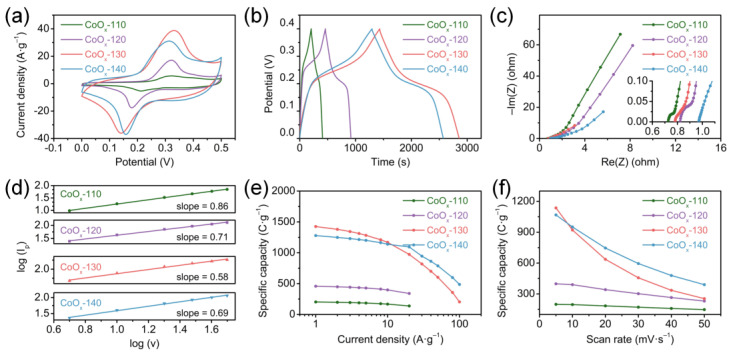
Electrochemical properties of the CoO_x_ electrodes: (**a**) the CV curves at 5 mV s^−1^, (**b**) the GCD curves at 1 A g^−1^, (**c**) Nyquist plots, (**d**) the linear plots of log (I_p_) versus log (ν), (**e**) specific capacities at various current densities, and (**f**) specific capacities at various scan rates.

Meanwhile, as the scanning speed increases, the oxidation and reduction peaks shift positively and negatively, respectively, which is related to the increase in the inescapable polarization effect inside the electrodes. Figure 4a compares the CV curves of all CoO_x_ electrodes., and a larger curve area represents a higher specific capacity. The calculated specific gravimetric capacities of the CoO_x_-110, CoO_x_-120, CoO_x_-130, and CoO_x_-140 are 198.5, 398, 1136, and 1068 C g^−1^, respectively. The specific capacity is related closely to the morphology, composition, and surface characteristics of the material. In addition, all the CV curves provide a power-law relationship between peak currents and scan rates (I_p_ = aν^b^), and the electrochemical kinetic behavior of the electrode is determined by calculating the b value according to the previously reported method [40], where b ≤ 0.5 is attributed to diffusion control, b ≥ 1 is ascribed to capacitance control, and 1 > b > 0.5 is synergistic control of those two. Figure 4d shows the plots of the log (I_p_) and log (ν) for all CoO_x_ electrodes, where the slopes are expressed as the b values. According to the fitting results in Figure S2 (black), the b values of the CoO_x_-110, CoO_x_-120, CoO_x_-130, and CoO_x_-140 are 0.86, 0.71, 0.58, and 0.69, respectively. All the CoO_x_ electrodes show a synergy of diffusion and capacitance control; the CoO_x_-130 is extremely inclined to diffusion control. Therefore, the current at a certain potential can be expressed as the sum of the following two parts: diffusion control (k_2_ν^1/2^) and capacitance control (k_1_ν) [41]. Figure S2 (red) compares the capacitance contribution rates of CoO_x_ electrodes at 5 mV s^−1^. Obviously, the CoO_x_-130 has the lowest capacitance contribution under the same scan rate, which confirms that its battery characteristics are the strongest, in agreement with the result of the b value.

**Figure 5:**
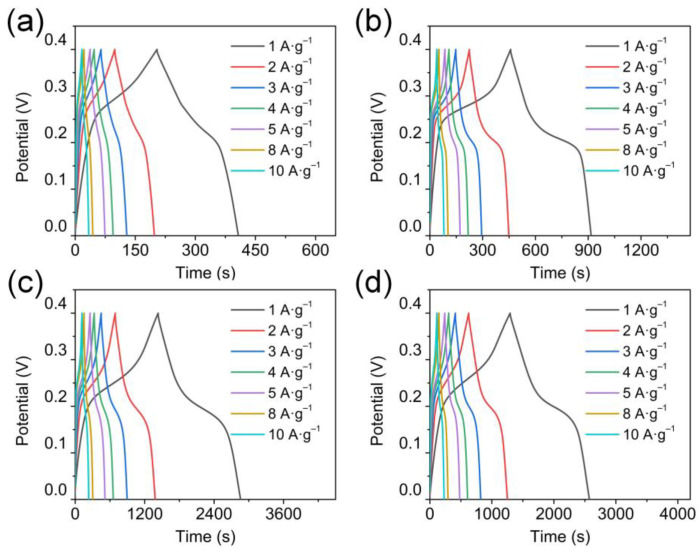
GCD curves of (**a**) CoO_x_-110, (**b**) CoO_x_-120, (**c**) CoO_x_-130, and (**d**) CoO_x_-140 at various current densities.

Figure 5a–d shows the GCD curves of the CoO_x_ electrodes from 1 to 10 A g^−1^ (0–0.4 V). Consistent with the CV results, platforms representing the characteristics of pseudocapacitors are observed in all GCD curves. The specific capacity of the CoO_x_-130 nanowires is 1425 C g^−1^ (3562.5 F g^−1^ at 1 A g^−1^), calculated from the GCD curves in Figure 4b, which is much higher than those of CoO_x_-110 (203 C g^−1^), CoO_x_-120 (457.5 C g^−1^), and CoO_x_-140 (1278.5 C g^−1^). The following three aspects give reasons for the ultrahigh capacity of the as-prepared CoO_x_-130: (1) large specific surface area and homogeneous mesopore distribution; (2) appropriate relative content of Co_3_O_4_ and CoO; (3) the aspect ratio of nanowires is more favorable for ion transport and electron conduction. In addition, as the current density increases, the capacity shows a slow decline (Figure 4e) due to the low utilization rate of electrode materials. However, the CoO_x_-130 electrode still maintains 1169 C g^−1^ (82% retention) at 10 A g^−1^, while only 168, 398, and 1138.5 C g^−1^ for CoO_x_-110, CoO_x_-120, and CoO_x_-140, respectively. Notably, the specific capacity of the CoO_x_-130 decreases sharply when the current density is greater than 10 A g^−1^, while the CoO_x_-140 decreases gently and remains at 38% compared to 1 A g^−1^. The reason may be that electrons are generated faster at high current density, and the short rods in the CoO_x_-140 electrode can shorten the electron transport path, thereby providing exceptional capacity performance at high current density. As the scanning rate increases in Figure 4f, the CoO_x_-140 shows better rate performance, which is in agreement with the results in Figure 4e. The impedance behavior of the CoO_x_ electrode is investigated by testing the EIS, and the results are displayed in Figure 4c. Obviously, all EIS spectra consist of a semicircle and a straight line. Equivalent series resistance (R_s_), including the internal resistance of the CoO_x_, surface contact resistance between CoO_x_ and NF, and the resistance of electrolytes, is determined by the intercept on the X-axis. Charge transfer resistance (R_ct_) at the electrode/electrolyte interface is determined by the semicircle diameter [42]. As the frequency decreases, the double-layer contribution of the electrode diminishes gradually. Additionally, the charge transfer turns to the material transfer dominant, where an inflection point appears on the curve. The slope of the following linear part determines the Warburg impedance (Z_w_) [43]. The higher the slope, the more likely the redox reaction will occur. Based on the equivalent circuit in the inset of Figure 5c, the values of R_s_ for CoO_x_-110, CoO_x_-120, CoO_x_-130, and CoO_x_-140 are 0.72, 0.81, 0.77, and 0.97 Ω, respectively, and the values of R_ct_ are 0.07, 0.17, 0.05, and 0.08 Ω, accordingly. The CoO_x_-130 manifests the lowest R_ct_, which is probably due to the better crystallinity, an appropriate mass ratio of Co_3_O_4_/CoO, and a desirable porous structure.

**Figure 6:**
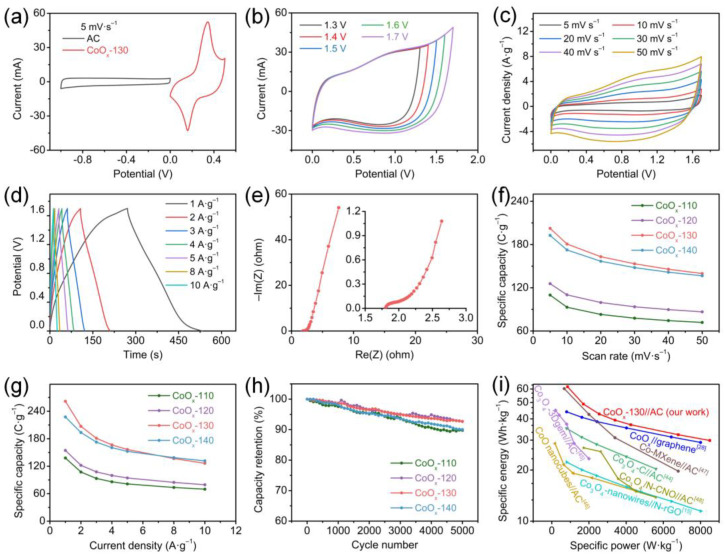
(**a**) The window match of CoO_x_-130 (red) and AC (black); (**b**) CV curves at various potential windows at 50 mV s^−1^; (**c**) CV curves at various scan rates, (**d**) GCD curves at various current densities, and (**e**) Nyquist plot of the CoO_x_-130//AC HSC; (**f**) specific capacities at different scan rates, (**g**) specific capacities at different current densities, and (**h**) cycling performance of the CoO_x_//AC HSCs; (**i**) Ragone plots for the CoO_x_-130//AC HSC.

CoO_x_ cathode and commercial AC anode are used to assemble the HSCs. The CV curves at different scan rates of AC present a rectangle shape in Figure S3a, indicating typical capacitive behavior. The GCD curves in Figure S3b are symmetrical and highly linear under different current densities, which is also considered a representative characteristic of electric double-layer capacitors and agrees with the CV results. It is found that the AC displays a low R_s_ of 0.82 Ω (Figure S3c). According to the curves of GCD (1 A g^−1^) and CV (5 mV s^−1^), their gravimetric specific capacitances are 225.3 and 180 F g^−1^, respectively. Notably, the AC electrode show superb rate capability and cyclic stability, referring to the capacitance retention ratios of 87.8% (from 5 to 50 mV s^−1^, Figure S3d), 72.4% (from 1 to 10 A g^−1^, Figure S3e), and 90.1% (after 10,000 cycles at 5 A g^−1^, Figure S3f). The CoO_x_-130 electrode and AC electrode exhibit complementary current responses, as shown in Figure 6a. A steady working window is obtained from 0 to 1.7 V for HSCs by testing various potential windows in Figure 6b. CV curves of the CoO_x_-130//AC HSC shows mild redox peaks and distorted rectangles at different scan rates in Figure 6c, indicating the battery and capacitive characteristics of the HSC. The GCD curves of the CoO_x_-130//AC HSC at a series of current densities are shown in Figure 6d, and the mild plateaus during charging or discharging, especially at low current densities, indicate a combination of battery-type CoO_x,_ and capacitive AC. The impedance plot of the CoO_x_-130//AC HSC is shown in Figure 6e, which shows low R_s_ and R_ct_ values of 1.8 Ω and 0.27 Ω, respectively. The specific capacity of CoO_x_-130//AC HSC under 1 A g^−1^ is 261.8 C g^−1^, which is higher than those of CoO_x_-110 (138 C g^−1^), CoO_x_-120 (154.5 C g^−1^), and CoO_x_-140 (227.5 C g^−1^). The other CoO_x_ electrodes-based HSCs have also been tested, and the results are displayed in Figure S4. With increasing scan rate and current density, the specific capacities of all CoO_x_//AC HSCs are summarized in Figure 6f,g, respectively. At 10 A g^−1^, the specific capacity of CoO_x_-130//AC HSC still reaches up to 126 C g^−1^. Notably, the specific capacity of the CoO_x_-140//AC HSC exceeds that of CoO_x_-130//AC HSC at 8 A g^−1^, confirming its high-rate capability and is consistent with the results of electrode test.

Cyclic performance is a critical parameter to evaluate the practicability of the SCs. All CoO_x_//AC HSCs are tested repeatedly at 5 A g^−1^ from 0 to 1.7 V. With the progress of cycles, the specific capacity of HSCs decreases gradually in Figure 6h, and 92.72% of the primary specific capacity remains after 5000 cycles for the CoO_x_-130//AC HSC. The capacity retention of the CoO_x_-130//AC HSC is higher than those of CoO_x_-110//AC HSC (89.77%), CoO_x_-120//AC HSC (92.65%), and CoO_x_-140//AC HSC (90.04%), indicating the desirable properties of the CoO_x_-130 electrode.

As shown in Figure 6i, the CoO_x_-130//AC HSC in this work possesses an impressive specific energy of 61.8 Wh kg^−1^ at a specific power of 850 W kg^−1^ and can retain 29.9 Wh kg^−1^ at a high specific power of 8500 W kg^−1^, which is superior to many reported HSCs [15,28,44,45,46,47,48]. In addition, the gravimetric specific capacity, operating window, rate performance, and cycle stability of these devices are compared with reported HSCs in Table S2. It can be seen that the CoO_x_-130//AC HSC exhibits a high gravimetric specific capacity, large working window, good cycle stability, and comparable rate performance. Notably, the superior specific energy density of the CoO_x_-130//AC HSC can be ascribed to its ultrahigh gravimetric capacity and large operating window of 1.7 V. The outstanding capacity of the CoO_x_ cathode is derived from the large surface area, appropriate pore size distribution and Co_3_O_4_/CoO ratio, and desirable aspect ratio of nanowires. The aforementioned results indicate that the CoO_x_-130 nanowires can be an advanced cathode material in the field of SCs.

## 4. Conclusions

In this study, CoO_x_ nanowire electrodes with different aspect ratios are synthesized by hydrothermal reaction at different temperatures and post-annealing treatment. The CoO_x_-130 achieves an exceptional specific capacity of 1424.8 C g^−1^ due to its large specific surface area and appropriate mesoporous size. Notably, the CoO_x_-140 shows an excellent rate performance of 38% retention at 100 A g^−1^. In addition, the CoO_x_-130//AC HSC reaches an excellent specific capacity of 261.8 C g^−1^ and a specific energy of 61.8 Wh kg^−1^. Impressively, the capacity retains 92.72% after 5000 cycles at 5 A g^−1^, displaying exceptional cycle stability. Therefore, this study shows the great potential of the CoO_x_ nanowires for SCs application and offers an important reference for the fabrication of high-performance pseudocapacitive electrodes.

## Data Availability

Not applicable.

## Supplementary Materials

The following supporting information can be downloaded at: https://www.mdpi.com/2079-4991/13/4/749/s1, Figure S1: XRD patterns of the CoO_x_-130 precursor; Figure S2: b-value (black) and capacitance contribution (red) at 5 mV s^−1^ of CoO_x_ electrodes.; Figure S3: Electrochemical properties of the AC electrodes: (a) the CV curves at various scan rates, (b) the GCD curves at various current densities, (c) Nyquist plot, (d) the specific capacitances at different current densities, (e) the specific capacitances at different current densities, and (f) the cycling performance at 5 A g^−1^; Figure S4: CV curves at various scan rates: (a) CoO_x_-110//AC HSC, (b) CoO_x_-120//AC HSC, and (c) CoO_x_-140//AC HSC; GCD curves at various current densities: (d) CoO_x_-110//AC HSC, (e) CoO_x_-120//AC HSC, and (f) CoO_x_-140//AC HSC; Table S1: The quantitative analysis of CoO_x_ nanowire; Table S2: The electrochemical capability of CoO_x_-130//AC in comparison to CoO_x_-based materials previously reported.
