# Correction: Ji et al. Mesoporous Cobalt Oxide (CoO_x_) Nanowires with Different Aspect Ratios for High Performance Hybrid Supercapacitors. *Nanomaterials* 2023, *13*, 749

**DOI:** 10.3390/nano14050459

**Published:** 2024-03-01

**Authors:** Haomin Ji, Yifei Ma, Zhuo Cai, Micun Yun, Jiemin Han, Zhaomin Tong, Mei Wang, Jonghwan Suhr, Liantuan Xiao, Suotang Jia, Xuyuan Chen

**Affiliations:** 1 State Key Laboratory of Quantum Optics and Quantum Optics Devices, Institute of Laser Spectroscopy, Collaborative Innovation Center of Extreme Optics, Shanxi University, Taiyuan 030006, China; 2 Department of Polymer Science and Engineering, School of Mechanical Engineering, Sungkyunkwan University, Suwon 16419, Republic of Korea; 3 Faculty of Technology, Natural Sciences and Maritime Sciences, Department of Microsystems, University of Southeast Norway, N-3184 Borre, Norway

## Text Correction

There was an error in the original publication [1]. As the JCPDS Card 48-0083 is modified by new synchrotron X-ray powder diffraction data, the chemical composition and crystal structure of the CoO_x_-130 precursor require modification in the published article. A correction has been made to Section 3. Results and Discussion, Paragraph 2:

“Multiple diffraction peaks indicate that the composition of the precursor is complex, but the main component is certainly Co_6_(CO_3_)_2_(OH)_8_·H_2_O (JCPDS No.48–0083) [31]. Accordingly, the chemical reactions related to the precursors can be described as follows:

6Co^2+^ + 8OH^−^ + 2CO_3_^2−^ + H_2_O → Co_6_(CO_3_)_2_(OH)_8_·H_2_O”

## Citation Correction

In the original publication [1], Bhojane, P.; Bail, A.; Shirage P. A quarter of a century after its synthesis and with >200 papers based on its use, Co(CO_3_)0.5 30 (OH)·0.11H_2_O proves to be Co_6_(CO_3_)_2_ (OH)_8_·H_2_O from synchrotron powder diffraction data. *Acta Crystallogr. C Struct. Chem.* **2019**, *75*, 61–64. was not cited. The citation has now been inserted in Section 3. Results and Discussion, Paragraph 2, and replaced the original citation (ref. 31), Zhang, Z.; Hao, J.; Yang, W.; Lu, B.; Ke, X.; Zhang, B.; Tang, J. Porous Co_3_O_4_ Nanorods-Reduced Graphene Oxide with Intrinsic Peroxidase-Like Activity and Catalysis in the Degradation of Methylene Blue. *ACS Appl. Mater. Interfaces* **2013**, *5*, 3809–3815.

## Figure Correction in Supplementary Materials

Following the above, there was a mistake in Figure S1 of the original publication [1]. 

The authors state that the scientific conclusions are unaffected. This correction was approved by the Academic Editor. The original publication has also been updated.
