# Supplementary material for: Mesoporous Cobalt Oxide (CoOx) Nanowires with Different Aspect Ratios for High Performance Hybrid Supercapacitors
Source: Nanomaterials (Basel). 2023 Feb 16;13(4):749. doi: 10.3390/nano13040749 (PMC9966480; doi:10.3390/nano13040749)
Supplement: Supplementary file 1 [file nanomaterials-13-00749-s001.zip › nanomaterials-2203597-supplementary.pdf]

nanomaterials

Supplementary Materials

Mesoporous Cobalt Oxide (CoO_x) Nanowires with Different Aspect Ratios for High Performance Hybrid Supercapacitors

Haomin Ji [1], Yifei Ma [1,*], Zhuo Cai [1], Micun Yun [1], Jiemin Han [1], Zhaomin Tong [1], Mei Wang [1,*], Jonghwan Suhr [2], Liantuan Xiao [1], Suotang Jia [1] and Xuyuan Chen [1,3]

1 State Key Laboratory of Quantum Optics and Quantum Optics Devices, Institute of Laser Spectroscopy, Collaborative Innovation Center of Extreme Optics, Shanxi University, Taiyuan 030006, China; haominji@126.com (H.J.); caizhuo2229@163.com (Z.C.); yunmicun@163.com (M.Y.); jiemin.han@foxmail.com (J.H.); zhaomin.tong@sxu.edu.cn (Z.T.); xlt@sxu.edu.cn (L.X.); tjia@sxu.edu.cn (S.J.); xuyuan.chen@usn.no (X.C.)
2 Department of Polymer Science and Engineering, School of Mechanical Engineering, Sungkyunkwan University, Suwon 16419, Republic of Korea; suhr@skku.edu
3 Faculty of Technology, Natural Sciences and Maritime Sciences, Department of Microsystems, University of Southeast Norway, N-3184 Borre, Norway
* Correspondence: mayifei@sxu.edu.cn (Y.M.); wangmei@sxu.edu.cn (M.W.)

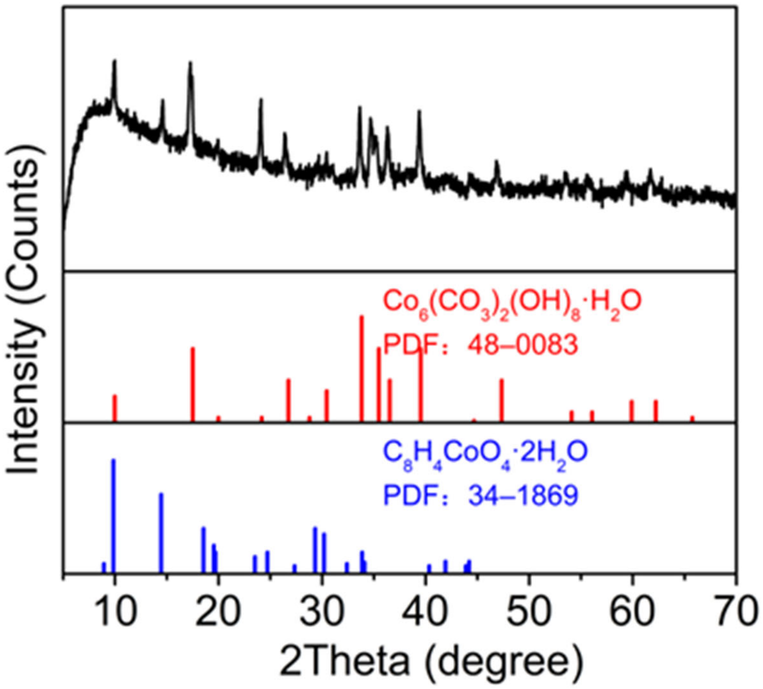

Figure S1. XRD patterns of the CoO_x-130 precursor.

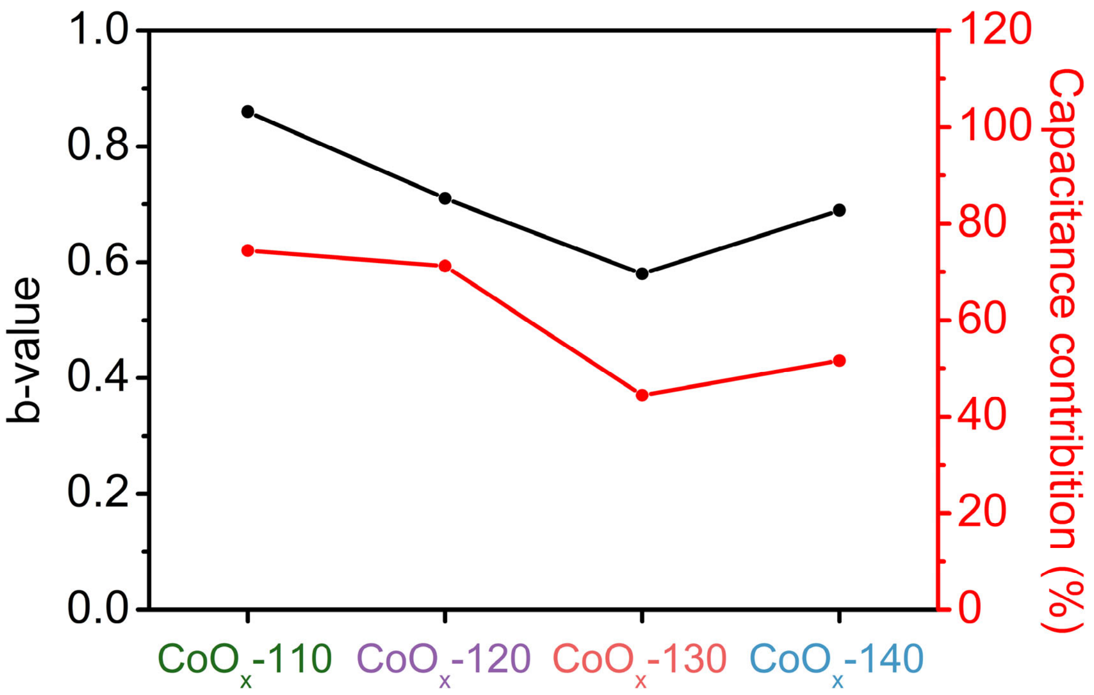

Figure S2. b-value (black) and capacitance contribution (red) at 5 mV s⁻¹ of CoO_x electrodes.

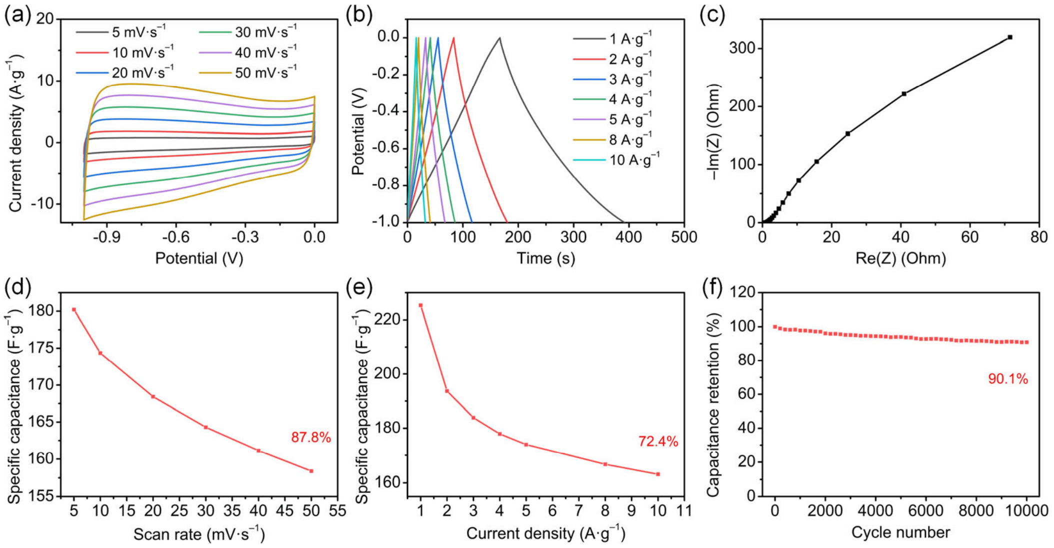

Figure S3. Electrochemical properties of the AC electrodes: (a) the CV curves at various scan rates, (b) the GCD curves at various current densities, (c) Nyquist plot, (d) the specific capacitances at different current densities, (e) the specific capacitances at different current densities, and (f) the cycling performance at 5 A g^{-1}.

nanomaterials

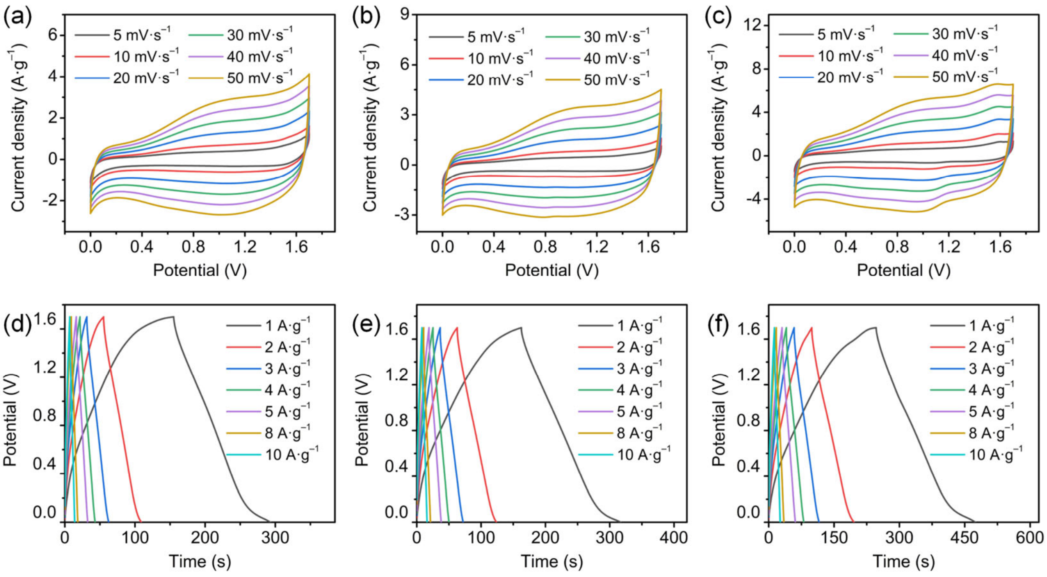

Figure S4. CV curves at various scan rates: (a) CoO_x-110//AC HSC, (b) CoO_x-120//AC HSC and (c) CoO_x-140//AC HSC; GCD curves at various current densities: (d) CoO_x-110//AC HSC, (e) CoO_x-120//AC HSC and (f) CoO_x-140//AC HSC.

Table 1. The quantitative analysis of CoO_x nanowires.

	Co_3O_4 (wt%)	CoO (wt%)
CoO_x-110	59.5	40.5
CoO_x-120	57.6	42.4
CoO_x-130	58.7	41.3
CoO_x-140	58.9	41.1

Table 2. The electrochemical capability of CoO_x-130//AC in comparison to repored CoO_x-based materials.

Materials	Specific capacity	Potential	Cyclic performance	Rate capability	Reference
CoO_x-130//AC	261.8 C g^{-1} at 1 A g^{-1}	0–1.7 V	92.72% after 5000 cycles at 5 A g^{-1}	48.12% from 1 A g^{-1} to 10 A g^{-1}	Our Work
Co_3O_4 nanowire//N-rGO	100 C g^{-1} at 1 A g^{-1}	0–1.6 V	93.3% after 10000 cycles at 2 A g^{-1}		15
CoO_x//graphene	198.24 C g^{-1} at 1 A g^{-1}	0–1.6 V	73.9% after 6000 cycles at 3 A g^{-1}	54.6% from 1 A g^{-1} to 15 A g^{-1}	28
Co_3O_4-C//AC	161.28 C g^{-1} at 1 A g^{-1}	0–1.6 V	82.6% after 10000 cycles at 5 A g^{-1}	65% from 1 A g^{-1} to 7 A g^{-1}	44
Co_3O_4-3Dgem//AC		0–1.6 V		51.9% from 0.5 A g^{-1} to 5 A g^{-1}	45
CoO nanocubes//AC		0–1.7 V			46
Co-MXene//AC	115.6 C g^{-1} at 0.5 A g^{-1}	0–1.5 V	93% after 1000 cycles at 5 A g^{-1}		47
Co_3O_4/N-CNO//AC	120.6 C g^{-1} at 2 A g^{-1}	0–1.8 V	78% after 2000 cycles at 6 A g^{-1}		48
